# Comparative proteomics of thylakoids from *Arabidopsis* grown in laboratory and field conditions

**DOI:** 10.1002/pld3.355

**Published:** 2021-10-20

**Authors:** Sarah E. Flannery, Federica Pastorelli, William H. J. Wood, C. Neil Hunter, Mark J. Dickman, Philip J. Jackson, Matthew P. Johnson

**Affiliations:** ^1^ Department of Molecular Biology and Biotechnology University of Sheffield Sheffield UK; ^2^ Department of Chemical and Biological Engineering University of Sheffield Sheffield UK

**Keywords:** acclimation, electron transport, light harvesting, photosynthesis, proteomics

## Abstract

Compared to controlled laboratory conditions, plant growth in the field is rarely optimal since it is frequently challenged by large fluctuations in light and temperature which lower the efficiency of photosynthesis and lead to photo‐oxidative stress. Plants grown under natural conditions therefore place an increased onus on the regulatory mechanisms that protect and repair the delicate photosynthetic machinery. Yet, the exact changes in thylakoid proteome composition which allow plants to acclimate to the natural environment remain largely unexplored. Here, we use quantitative label‐free proteomics to demonstrate that field‐grown Arabidopsis plants incorporate aspects of both the low and high light acclimation strategies previously observed in laboratory‐grown plants. Field plants showed increases in the relative abundance of ATP synthase, cytochrome *b*_6_ *f*, ferredoxin‐NADP^+^ reductases (FNR1 and FNR2) and their membrane tethers TIC62 and TROL, thylakoid architecture proteins CURT1A, CURT1B, RIQ1, and RIQ2, the minor monomeric antenna complex CP29.3, rapidly‐relaxing non‐photochemical quenching (qE)‐related proteins PSBS and VDE, the photosystem II (PSII) repair machinery and the cyclic electron transfer complexes NDH, PGRL1B, and PGR5, in addition to decreases in the amounts of LHCII trimers composed of LHCB1.1, LHCB1.2, LHCB1.4, and LHCB2 proteins and CP29.2, all features typical of a laboratory high light acclimation response. Conversely, field plants also showed increases in the abundance of light harvesting proteins LHCB1.3 and CP29.1, zeaxanthin epoxidase (ZEP) and the slowly‐relaxing non‐photochemical quenching (qI)‐related protein LCNP, changes previously associated with a laboratory low light acclimation response. Field plants also showed distinct changes to the proteome including the appearance of stress‐related proteins ELIP1 and ELIP2 and changes to proteins that are largely invariant under laboratory conditions such as state transition related proteins STN7 and TAP38. We discuss the significance of these alterations in the thylakoid proteome considering the unique set of challenges faced by plants growing under natural conditions.

## 1 INTRODUCTION

Most of our current understanding of developmental acclimation of photosynthesis in plants is based on studies performed under controlled laboratory conditions (reviewed by Schöttler & Tóth, 2014; Walters, 2005). In the model organism *Arabidopsis thaliana* (hereafter Arabidopsis) acclimation to low and high growth light intensities leads to distinct changes in the composition of the photosynthetic thylakoid membrane. Low light acclimation favoring increased amounts of light harvesting antenna complex II (LHCII) and photosystem I (PSI) to maximize solar energy capture, while high light acclimation leads to increases in the abundance of ATP synthase, cytochrome *b*_6_ *f* (cyt*b*_6_ *f*) and ferredoxin‐NADP^+^ reductase (FNR) complexes to maximize electron and proton transfer capacity to better utilize the available light (Bailey et al., 2001, 2004; Ballottari et al., 2007; Kouřil et al., 2013; Mikko et al., 2006; Schumann et al., 2017; Vialet‐Chabrand et al., 2017; Ware et al., 2015; Wientjes et al., 2013a, 2013b). However, Arabidopsis plants grown under natural field conditions show a very different phenotype to those grown under controlled laboratory conditions, differing substantially in thylakoid membrane protein composition and pigment content as well as leaf morphology (Mishra et al., 2012; Schumann et al., 2017; Wituszyńska et al., 2013). A key driver of these differences is that plants grown in the field are frequently exposed to multiple stresses including variable light, temperature and water availability in addition to the possibility of predation by other organisms (Atkin et al., 2006; Frenkel et al., 2008; Poorter et al., 2006; Ruban, 2015). These factors can affect the rate of damage to photosynthetic machinery, the rate of electron transport and demand for water, leading to decreased photosynthetic efficiency and lower crop yields (Li et al., 2009). Unsurprisingly therefore, various fitness‐related traits, such as seed size and germination rate, vary greatly in the field (Atwell et al., 2010; Brachi et al., 2010; Malmberg et al., 2005), while phenotypes associated with loss of many key photosynthetic regulatory proteins in Arabidopsis are only observed under naturally fluctuating light conditions (Frenkel et al., 2008; Külheim et al., 2002; Semchuk et al., 2009; Suorsa et al., 2012). Thus, while use of a constant light intensity, temperature and humidity in the laboratory growth chamber improves the reproducibility of results, it can also hinder our understanding of acclimation to the natural environment and limit information on how different protective mechanisms are integrated.

In recent years, substantial efforts have been made to better characterize the differences in thylakoid membrane protein composition and light harvesting and electron transfer function between laboratory and field grown Arabidopsis plants (Mishra et al., 2012; Schumann et al., 2017; Wituszyńska et al., 2013). Functional studies employing chlorophyll (Chl) fluorescence, absorption spectroscopy and infra‐red gas exchange analysis have shown that field grown plants generally show an increased capacity for CO_2_ assimilation, PSII electron transfer and PSII photoprotection through dissipation of excess absorbed solar energy by non‐photochemical quenching (NPQ) compared to laboratory grown plants, while the capacity for excitation energy input balancing between the PSI and PSII via state transitions was not significantly different (Mishra et al., 2012; Schumann et al., 2017; Wituszyńska et al., 2013). A combination of immunoblotting and absorption‐based spectroscopic assays has determined increases in the Chl *a*/*b* ratio, ATP synthase, cyt*b*_6_ *f*, PSI light harvesting protein 5 (LHCA5) and the photoprotective xanthophyll cycle pigments and PSBS protein abundance in field grown plants (Mishra et al., 2012; Schumann et al., 2017; Wituszyńska et al., 2013). Moreover, certain proteins were only observed under field conditions such as Early Light Inducible Proteins 1 and 2 (ELIP1 and 2) (Mishra et al., 2012). Yet these methods by their nature can only conveniently sample changes in a relatively small number of proteins. As an alternative approach, Wituszyńska et al. (2013) employed transcriptomics to identify genes undergoing altered expression in the field versus laboratory conditions, with increases seen for ELIP1 and decreases seen for LHCII components LHCB1.4, LHCB2.2, 2.4 and the minor monomeric antenna CP29 isoform LHCB4.2. However, since changes in gene expression do not necessarily translate into changes in protein abundance, these data need to be interpreted cautiously. Recently, mass spectrometry‐based proteomics was used to analyze acclimation to fluctuating laboratory light in Arabidopsis leaves (Niedermaier et al., 2020). However, no proteomic analysis has yet been employed to systematically study the thylakoid membrane of plants grown under field versus laboratory conditions.

Here, we address this gap in our knowledge, employing mass spectrometry to perform a label‐free quantitative proteomic comparison of the thylakoid membranes of outdoor (Field)‐ and laboratory (Lab)‐grown *A. thaliana* plants to further our understanding of acclimation and photoprotection in the thylakoid membrane. Our study highlights those proteins and regulatory mechanisms that are instrumental in the developmental adaptation of Arabidopsis to natural conditions, providing context for our previous work on acclimation to varying growth light intensity under laboratory conditions (Flannery et al., 2021).

## 2 MATERIALS AND METHODS

### 2.1 Growth and acclimation of Arabidopsis

**FIGURE 1:**
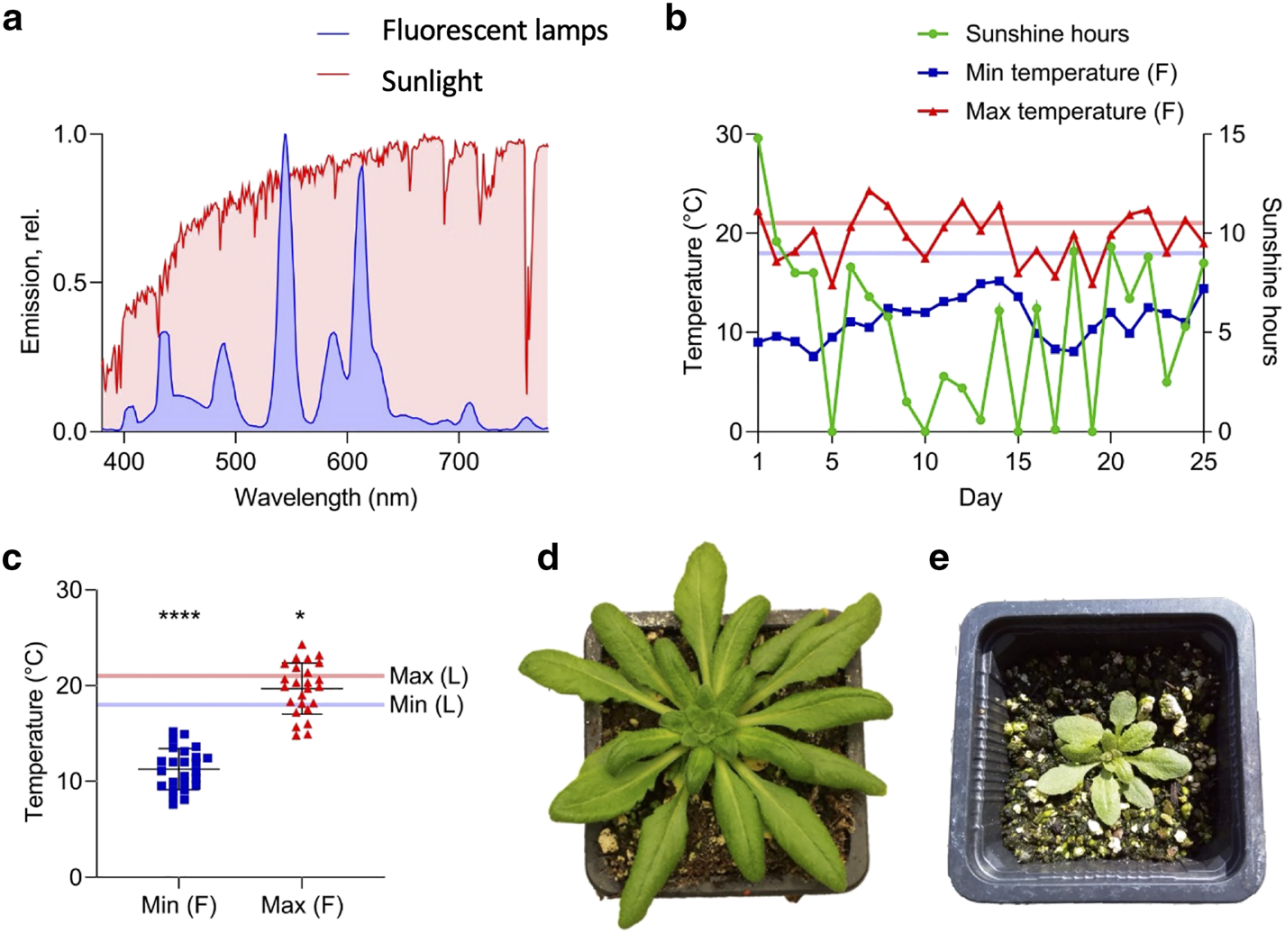
Characterization of light, temperature conditions and growth for Arabidopsis plants cultivated in the lab and field. (a) Spectral composition of natural sunlight recorded under field conditions (red) and from fluorescent lamps for laboratory conditions (blue). (b) Weather data (provided by Weston Park weather station, Sheffield, UK) in the form of daily maximum temperature, minimum temperature and hours of sunshine for the 25‐day period from 21 May to 14 June 2018. Sunshine hours were defined as the number of hours during that day in which the light intensity exceeded 120 W/m^2^. The pale red and blue lines indicated the daytime and nighttime temperatures, respectively, of the growth chamber for comparison. (c) Minimum and maximum daily temperatures experienced by field (F) Arabidopsis compared to lab (L) Arabidopsis (pale blue and red lines). Asterisks indicate significance from two‐tailed one‐sample t‐tests comparing minimum field temperature to minimum lab temperature (****P < .0001) and maximum field temperature to maximum lab temperature (*P < .05). (d and e) Representative images of Lab‐ and Field‐grown Arabidopsis plants respectively

*A. thaliana* plants (Col‐0) (15 per light condition) were grown on John Innes M3 compost (4 parts) mixed with perlite, and vermiculite (1 part each). Growth was started in a Conviron plant growth room under fluorescent bulbs (emission spectrum shown in Figure 1a) at 60% relative humidity, 21°C daytime, 18°C nighttime temperatures, at a light intensity of 150‐μmol photons m^−2^ s^−1^ with a 12‐h photoperiod. Light intensity was measured as photosynthetically active radiation (PAR) on a LI‐190 light meter. After 2 weeks, or until rosettes reached a diameter of around 3 cm, plants were transferred to either a controlled environment growth chamber or to an outdoor growth facility (Arthur Willis Environment Facility, University of Sheffield, 53°22′54.4″N 1°29′56.2″W). Plants were acclimatized for different lengths of time prior to harvesting to account for variable maturation rate depending on day length and light intensity (Cho et al., 2017). Local weather data for the acclimation period of outdoor‐grown plants was provided by the Weston Park Weather Station, Sheffield, which recorded minimum and maximum temperatures of each day along with sunshine hours. Sunshine hours were defined as the number of hours per day at which the light intensity exceeded 120 W/m^2^. A conversion factor 1 W/m^2^ = 4.57 μmol photons m^−2^ s^−1^ (Thimijan & Heins, 1983) was applied.

### 2.2 Electron microscopy of leaf thin sections

Leaf discs of 1‐cm diameter were taken at the point of harvest from positions in the center of exposed leaves. Electron micrographs of leaf thin sections were obtained according to Wood et al. (2018).

### 2.3 Structured illumination microscopy

Samples from leaves were prepared, imaged and analyzed according to Wood et al. (2019).

### 2.4 Isolation of thylakoid membranes

Thylakoid membranes were isolated according to Albertsson et al. (1994) with the addition of 10‐mM NaF to all buffers.

### 2.5 Chlorophyll analysis

Absorption spectra were taken on an Agilent Technologies Cary 60 UV–VIS spectrophotometer. Chlorophyll concentration and chlorophyll *a* to *b* ratios were determined according to Porra et al. (1989).

### 2.6 BN‐PAGE

Stromal lamellae were solubilized at .5‐mg/ml Chl in 2% digitonin, 50‐mM Bis Tris pH 7.2, 10‐mM NaF, 10% glycerol, for 1 h on ice. Grana membranes were solubilized in 1.0% *n*‐dodecyl α‐D‐maltoside, 50‐mM Bis‐Tris pH 7.2, 10‐mM NaF, 10% glycerol, for 1 h on ice. Solubilized protein complexes were isolated and separated by BN‐PAGE, as previously described (Wood et al., 2019), before Coomassie staining and imaging.

### 2.7 Low‐temperature fluorescence spectroscopy

77 K fluorescence spectroscopy was carried out as previously described (Wood et al., 2019).

### 2.8 Thylakoid membrane protein extraction and proteolytic digestion

Thylakoid membranes were solubilized by sonication in 1% (w/v) sodium laurate as described previously (Lin et al., 2013). Starch granules were then removed by centrifugation at 10,000 *x g* for 2 min. Aliquots of the supernatant containing 50‐μg protein (Bio‐Rad DC assay) were adjusted to 15 μl with 1% (w/v) sodium laurate, 100‐mM triethylammonium bicarbonate (TEAB) pH 8.5 then reduced by the addition of 1.5‐μl 100‐mM tris(2‐carboxyethyl)phosphine‐HCl and incubation at 37°C for 30 min. Proteins were S‐alkylated by the addition of 1.5 μl of 200 mM iodoacetamide in 100 mM TEAB pH 8.5 and incubation at ambient temperature in the dark for 30 min. Samples were adjusted to 50 μl with 1% (w/v) sodium laurate, 100‐mM TEAB pH 8.5 and proteolytic digestion was carried out after the addition of 2‐μg pre‐mixed trypsin/endoproteinase Lys‐C (Promega) and incubation for 3 h at 37°C. Extraction of sodium laurate was performed as previously described (Lin et al., 2013) by adding an equal volume of ethyl acetate and acidification with 10 μl 10% (v/v) trifluoroacetic acid (TFA). The samples were vortexed for 1 min then centrifuged at 15,700 *x g* for 5 min to accelerate phase separation. The peptide‐containing lower phase was isolated, dried by vacuum centrifugation and dissolved in 50 μl 0.5% (v/v) TFA, 3% (v/v) acetonitrile before desalting with C18 spin columns (Thermo Scientific) according to the manufacturer's protocol. The peptides were again dried by vacuum centrifugation and stored at −20°C.

### 2.9 Analysis by mass spectrometry and protein identification

Peptides were dissolved in .5% (v/v) TFA, 3% (v/v) acetonitrile and 400 ng of each of three biological replicates were analyzed in triplicate in randomized order. Peptides were resolved on an EASY‐Spray PepMap RSLC C_18_ column (Thermo Scientific, 50 cm × 75 μm ID, 2 μm, 40°C) with the following gradient profiled delivered at 300 nl/min by a Dionex RSLCnano chromatography system (Thermo Scientific): 97% solvent A (.1% formic acid in water) to 10% solvent B (.08% formic acid in 80% acetonitrile) over 5 min, then 10% to 50% solvent B over 3 h. Mass spectrometry analysis was performed using a Q Exactive HF hybrid quadrupole‐Orbitrap (Thermo Scientific) using data dependent acquisition with profile full MS scans at 120,000 resolution and a maximum of 10 centroid product ion scans at 30000 resolution per cycle as per Flannery et al. (2021). Proteins were identified by searching the MS data files against the *A. thaliana* reference proteome database (http://www.uniprot.org/proteomes/UP000006548, downloaded on 10 December 2018) using MaxQuant v. 1.6.3.4 (Cox & Mann, 2008) with the intensity‐based absolute quantification (iBAQ) (Cox & Mann, 2008; Schwanhäusser et al., 2011) option selected. Search parameters were: carbamidomethyl‐Cys (fixed modification), Met oxidation, protein N‐terminal acetylation, Lys acetylation and Gln to pyro‐Glu conversion (variable modifications) with a maximum of two missed cleavages.

### 2.10 Mass spectrometry‐based protein quantification

Quantification results in the form of iBAQ (Cox & Mann, 2008; Schwanhäusser et al., 2011) intensities, as generated by MaxQuant (Cox & Mann, 2008) for the identified proteins, were processed using Perseus v. 1.6.2.3 (Tyanova et al., 2016). To compensate for variation due to sample loading and MS spectral acquisition timing, iBAQ intensities for the target proteins were normalized to the intra‐analysis sum of iBAQ intensities of key photosynthetic complexes PSII (PSBA, PSBB, PSBC, PSBD, PSBE, PSBF, PSBH, PSBO1, PSBO2, PSBP1, PSBP2, PSBQ1, PSBQ2, PSBR), PSI (PSAA, PSAB, PSAC, PSAD, PSAE1, PSAE2, PSAF, PSAG, PSAH, PSAK, PSAL, PSAN, PSAO), cyt*b*_*6*_ *f* (PETA, PETB, PETC, PETD), and ATP synthase (ATPA, ATPB, ATPC, ATPD, ATPE, ATPF, ATPH, and ATPI). Normalized iBAQ intensities for each MS analysis are provided in Table S2. The significance of changes in protein expression following acclimation to Lab or Field growth conditions was determined using a modified Welch's *t* test as implemented in Perseus (Tyanova et al., 2016). Protein identifications were assigned as being associated with the thylakoid membrane, lumen or plastoglobules using SUBA4 (Hooper et al., 2017). As discussed in Flannery et al. (2021), relative quantification based on normalization to equal amounts of chlorophyll may not give a realistic picture of changes in protein abundance when the ratio of protein to chlorophyll changes significantly. Indeed, as in plants acclimated to high light intensity in a controlled environment, Field thylakoids have an increased amount of protein relative to chlorophyll (see Section 3.2).

## 3 RESULTS

### 3.1 Field‐grown Arabidopsis experienced light and temperature conditions dramatically different to those grown in the lab

Arabidopsis seedlings were grown for 2 weeks in a controlled environment at a moderate light intensity (150 μmol photons m^−2^ s^−1^) under fluorescent artificial lighting (Figure 1a). Plants were subjected to 12 h of light per day with a daytime temperature of 21°C and a night time temperature of 18°C (Figure 1b,c). Following this 2‐week period, plants were either maintained for a further 3 weeks in the growth chamber (Lab) or moved outdoors (Arthur Willis Environment Centre, University of Sheffield, UK, 53°22′54.4″N 1°29′56.2″W) (Field). The Field plants were positioned such that there was minimal shading of sunlight from buildings or other structures so that the intensity of sunlight reaching the plants would be more representative of the weather conditions and of the gradual increases and decreases in light intensity of the day/night cycle. The emission spectra of the fluorescent lights in the growth chamber and of sunlight are shown in Figure 1a. The spectrum of sunlight was broader and more consistent across a wide range of wavelengths than that of the fluorescent lamps. In particular, sunlight showed a much greater relative emission at the longer wavelengths, around the far‐red 700–750 nm region, which preferentially excites PSI (reviewed in Johnson & Wientjes, 2020). Both Lab and Field grown plants were watered regularly to avoid drought stress, and pesticide‐free measures were taken to reduce predation of the Field plants by slugs and snails e.g. using copper tape around the outside of the trays and positioning above ground level.

The Field plants were grown outdoors for a 25‐day period from 21 May to 14 June 2018 before harvesting. Daylight lasted for 16–17 h at this location at the time of year the experiments were carried out. Weather data for this period, provided by the Weston Park Weather Station, Museums Sheffield, are shown in Figure 1b. The data recorded by the weather station consists of daily minimum temperature, maximum temperature, and sunshine hours, defined as the number of hours per day in which the light intensity exceeded 120 W/m^2^. With the conversion of 1 W/m^2^ = 4.57 μmol photons m^−2^ s^−1^ (Thimijan & Heins, 1983), this means that “sunlight hours” were those that exceeded 548 μmol photons m^−2^ s^−1^—much higher than in the growth chamber. The Field plants were exposed to a light intensity exceeding this value on all but 4 of the days, and on one day were exposed to 14.8 h of sunshine. This means that, overall, the Field plants consistently experienced increased light intensities compared to the Lab plants, in addition to a longer day length. The outdoor temperature was also highly variable compared to the controlled environment (Figure 1b,c). While the temperature of the growth chamber only varied by 3°C, on the hottest day outdoors there was a difference of 13.8°C between the minimum and maximum temperature. On average, both the maximum and the minimum temperatures outdoors were significantly lower than those of the growth chamber (Figure 1c). The combination of high light intensity and low temperature is particularly stressful for photosynthesis (Franklin et al., 2014; Ivanov et al., 2012; Öquist & Huner, 1993; Osmond, 1981; Savitch et al., 2002; Wanner & Junttila, 1999). High light intensity causes a build‐up of excitation energy, while low temperature reduces the rate of the reactions of the Calvin‐Benson‐Bassham (CBB) cycle, decreasing the electron sink capacity such that NADP^+^ is regenerated less efficiently. The result is the overreduction of PSI and PSII leading to formation of reactive oxygen species and photo‐oxidative stress (Li et al., 2009). Qualitatively, Field plants showed dramatic morphological differences compared to Lab plants (Figure 1d,e), with fewer, smaller and more curled leaves, as previously reported (Mishra et al., 2012; Schumann et al., 2017).

### 3.2 Field grown Arabidopsis plants show a smaller PSII and PSI antenna size, lowered PSI/PSII fluorescence emission ratio and lack the PSI‐LHCI‐LHCII supercomplex

**FIGURE 2:**
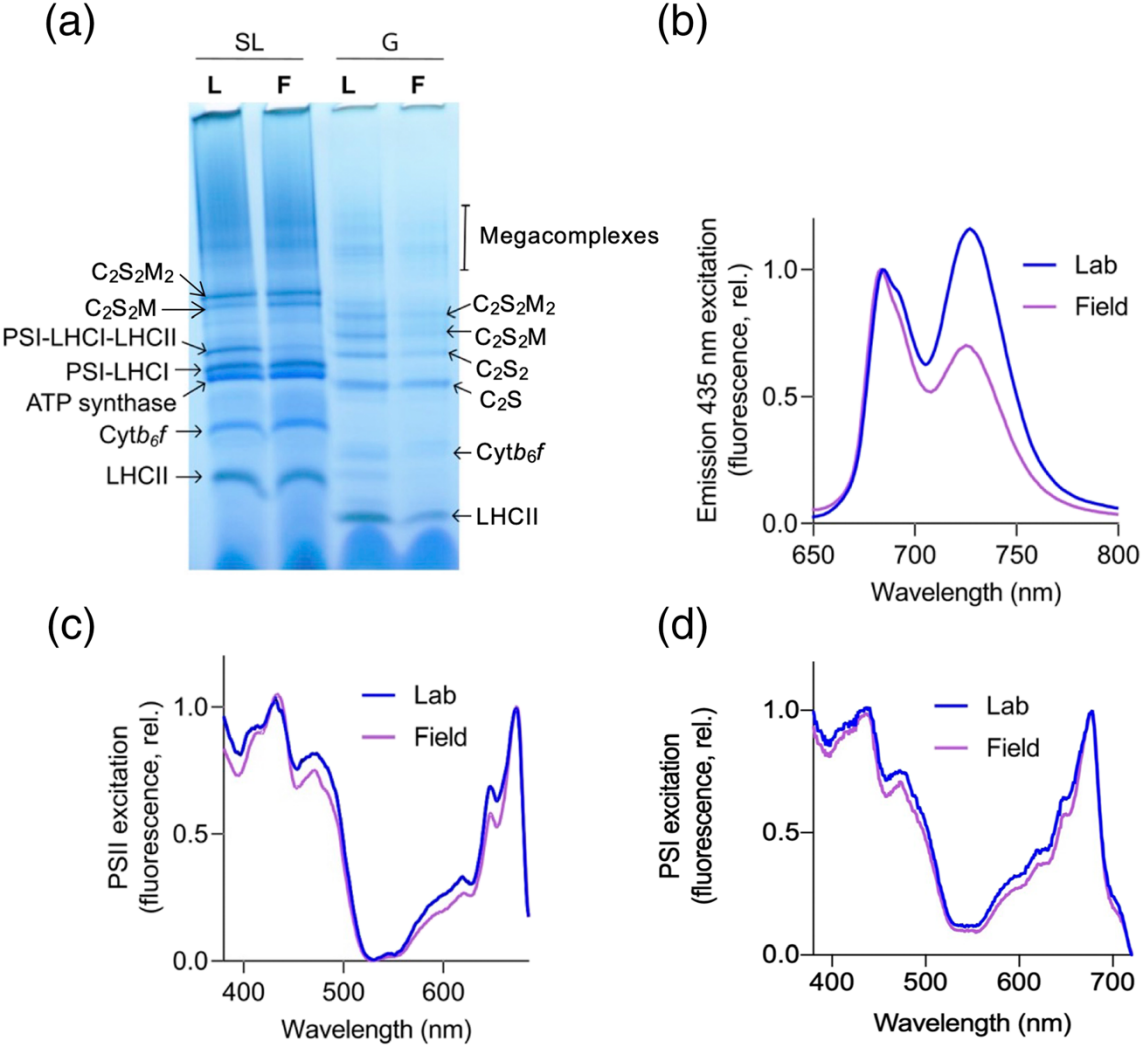
Characterization of thylakoid membrane protein complexes from Arabidopsis plants cultivated in the lab and field. (a) BN‐PAGE of solubilized complexes stromal lamellae (SL) and granal (G) thylakoid fractions from Lab (L) and Field (F) plants. (b) 77 K fluorescence emission spectra of Lab (blue) and Field (purple) thylakoids at 435 nm excitation. (c) 77 K 695‐nm fluorescence excitation spectra of PSII from Lab (blue) and Field (purple) thylakoids. (d) 77 K 735 nm fluorescence excitation spectra of PSI from Lab (blue) and Field (purple) thylakoids

Thylakoid membranes were isolated from leaf tissue pooled from at least 15 Lab or Field Arabidopsis plants. Despite clear phenotypic differences, calculated ratios of Chl *a* to *b* were very similar, 3.13 ± .03 for Lab versus 3.01 ± .02 for Field. However, this similarity does not necessarily indicate a similar antenna size, since Chl *a/b* ratios are affected by both antenna size and by the PSI/PSII ratio. Previous analyses have found an increase in the relative amount of Chl *a* in natural light compared to a controlled environment with a moderate light intensity (Mishra et al., 2012; Schumann et al., 2017). The ratio of protein to Chl was found to be considerably higher in the Field plants at 7.19 ± .18 versus 5.26 ± .31 for Lab plants. Analysis of the thylakoid membranes by BN‐PAGE revealed marked differences in the composition of the major photosynthetic complexes of the thylakoid membrane and in their distribution between the grana and stromal lamellae (Figure 2a). We first used digitonin to solubilize the stromal lamellae region of the thylakoid membrane on an equal chlorophyll basis, which revealed increases in the amounts C_2_S_2_M PSII‐LHCII supercomplex, cyt*b*_*6*_ *f* and ATP synthase complexes in the Field compared to Lab plants. The other major difference in stromal lamellae composition was the amount of the PSI‐LHCI‐LHCII supercomplex, which was virtually absent in the Field plants, but present in the Lab plants. The grana fraction that remains unsolubilised in digitonin was then solubilized in 1% *n*‐dodecyl α‐D‐maltoside (Figure 2a). Here a reduction in the number of “free” or L‐type LHCII trimers was observed in the Field plants, in addition to the C_2_S_2_M_2_, C_2_S_2_M, and C_2_S_2_ PSII‐LHCII supercomplexes (where L, M, and S denote loosely, moderately and strongly bound LHCII trimers to the PSII core dimer C_2_ respectively). However, the apparent abundance of the C_2_S supercomplex was similar. The near absence of the PSI‐LHCI‐LHCII supercomplex in the Field thylakoids was consistent with the lower ratio of fluorescence emission from PSI relative to PSII at 77 K (Figure 2b). Indeed, the PSI emission is dominant over PSII in the Lab grown plants while the opposite is true in the Field plants. The PSII and PSI fluorescence excitation spectra of Field thylakoids showed a lower contribution of Chl *b* wavelengths at 650 and 470–485 nm compared to the Lab grown plants consistent with a smaller antenna cross‐section (Figure 2c,d). While a decrease in the contribution of the long wavelength >700 nm forms to the PSI excitation spectrum was also seen, suggesting a smaller contribution from LHCI (Figure 2d).

### 3.3 Proteomic analysis of Arabidopsis grown under field conditions reveals changes in the abundance of key photosynthetic complexes and the antenna protein composition of PSI and PSII

**FIGURE 3:**
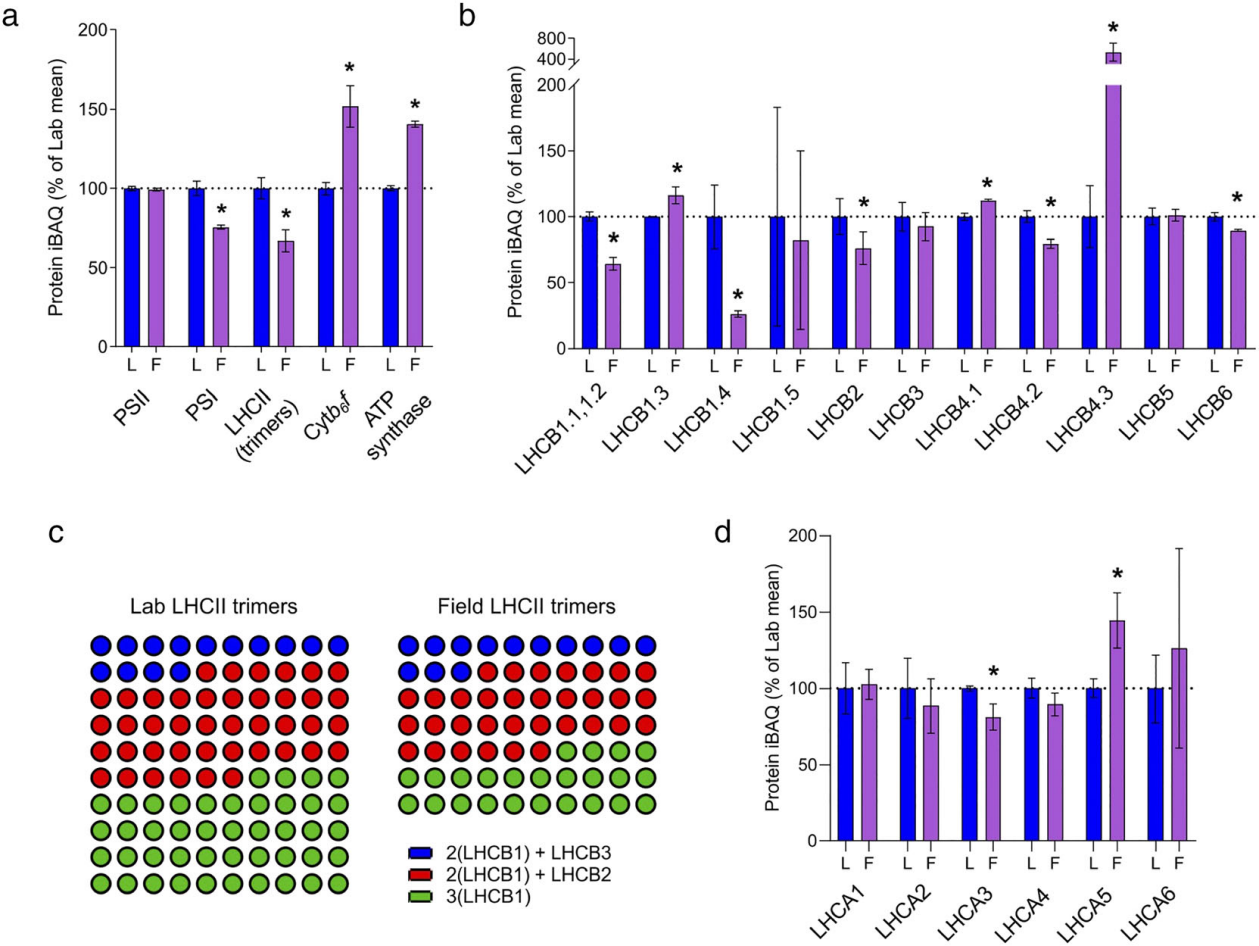
MS‐based quantification of light harvesting proteins and photosynthetic complexes from Arabidopsis plants cultivated in the lab and field. (a) Relative abundances in Lab (L) and Field (F) thylakoids of PSII, PSI, LHCII, cyt*b*_6_ *f*, and ATP synthase, expressed as a percentage of the mean Lab iBAQ values. The bars represent the means of three independent peptide preparations (*n* = 3), derived from pooled thylakoid samples, which were subject to MS analysis in triplicate in a randomized order. Error bars indicate mean ± SD. Significant differences between conditions were determined by a modified Welch's *t* test (*q < .05). (b) Relative abundances of LHCII subunits, with results represented as in (a). (c) Comparison of the numbers of LHCII trimers, shown as dots, in Lab and Field thylakoids and the distribution of trimer types. The number of trimers in Lab thylakoids is set to 100, and the trimers are categorized into trimers containing LHCB3 (blue dots), LHCB2 (red dots), or only LHCB1 (green). Abundance values for all LHCB1 isoforms (LHCB1.1, 1.2, 1.3, 1.4, and 1.5) were summed. (d) Relative abundances of LHCI isoforms, with results represented as in (a)

Thylakoid membranes from Lab and Field plants were prepared for proteomic analysis in triplicate by solubilization in 1% sodium laurate and digestion with trypsin/eLysC. Desalted peptides were analyzed by nanoLC‐MS/MS in triplicate with data dependent acquisition. MS data were searched against the UniProtKB proteome database to identify and quantify a total of 2926 proteins across both conditions, of which 460 were identified as being thylakoid‐associated. Relative quantification of proteins from MS data was performed using iBAQ values (Cox & Mann, 2008; Schwanhäusser et al., 2011) normalized to the intra‐analysis sum of proteins from the key photosynthetic complexes PSII, PSI, cyt*b*_*6*_ *f* and ATP synthase (Figure 3a) as described in Flannery et al., 2021. The normalized iBAQ values of the major photosynthetic complexes are presented in Figure 3 and displayed with the mean Lab value set to 100% for comparison. Consistent with data from plants acclimated to constant light intensity (Flannery et al., 2021) and with a previous study of natural light Arabidopsis by Schumann et al., 2017, the abundance of PSII remained at a constant level relative to the other key photosynthetic complexes (Figure 3a). Field plants had 25% less PSI compared to those grown in the lab, similar to results reported previously (Schumann et al., 2017), while the decrease shown by the PSI emission peak at 735 nm was ~40% (Figure 2b). This mismatch probably results from the absence of the PSI‐LHCI‐LHCII supercomplex, as seen in the BN‐PAGE analysis (Figure 2a), with an accompanying loss of energetically connected LHCII trimers (Figure 2d). Previously, downregulation of antenna proteins has been observed in Arabidopsis grown outdoors (Wituszyńska et al., 2013). Consistent with this, and with the reduction in the Chl *b* contribution to the PSII excitation spectra (Figure 2c), MS analysis revealed a 30% decrease in the relative abundance of LHCII trimers in Field thylakoids (Figure 3a). The extent of this decrease was two‐fold greater than that observed in thylakoids from plants acclimated to constant high light (Flannery et al., 2021), which was approximately 15%. This dramatic reduction in antenna size of the Field plants appears to be contradicted by the Chl *a*/*b* ratio, which did not change substantially. However, the reduction in the relative amount of Chl *b* in the antenna may be mitigated by the 25% decrease in PSI, enriched in Chl *a* (Figure 3a). The MS data show that the relative abundance of cyt*b*_*6*_ *f* increases by 50% in Field plants—substantially more than the 20% increase seen in plants acclimated to high light in the laboratory (Flannery et al., 2021) and contrary to the 16% decrease described previously by Schumann et al. (2017) (Figure 3a).

Quantitative proteomic analysis indicated varying behaviors of different LHCII constituent isoforms, shown in Figure 3b, in both trimeric and monomeric antenna proteins in the Field plants. Of the five LHCB1 isoforms in the Arabidopsis genome (Pietrzykowska et al., 2014), LHCB1.1 and LHCB1.2 could not be distinguished from one another because they have identical amino acid sequences but LHCB1.3, LHCB1.4, and LHCB1.5 were quantified separately. While the relative abundance of LHCII trimers decreased by 30% and there were substantial decreases in the relative abundance of LHCB1.1/1.2 (40%), LHCB1.4 (80%), and LHCB2 (25%), the LHCB1.3 isoform increased by 20% (Figure 3b). Consistent with previous analysis comparing Field and Lab plants (Mishra et al., 2012) and to its behavior under constant light acclimation described in Flannery et al., 2021, the relative abundance of LHCB3, which is only present in the LHCII M‐trimers (Caffarri et al., 2009), did not change. This could suggest that the reduction in the amount of LHCII arises mostly from fewer L‐trimers. However, LHCB6 (CP24), which links M‐trimers to the PSII core via LHCB3, showed a small decrease of around 10% in Field plants, while LHCB5 (CP26) remained constant (Figure 3b). Isoforms of another monomeric antenna protein associated with PSII, LHCB4 (CP29), also underwent some stoichiometric changes because of outdoor acclimation. For this protein, a 20% decrease in the relative abundance of the LHCB4.2 isoform was countered by increases in LHCB4.1 (10%), and LHCB4.3 (400%) (Figure 3b). The latter lacks the stromal C‐terminal domain, which interacts with both M‐trimers and LHCB6 (Pagliano et al., 2014) and mediates the interaction of PSII supercomplexes between granal membrane layers (Albanese et al., 2020). The 10% increase in LHCB4.1 in the Field thylakoids is comparable to the response of this isoform to constant low light acclimation (Flannery et al., 2021).

Differences in the composition of the LHCII trimers calculated from the MS data are shown in Figure 3c as color‐coded dots. Each dot represents one trimer, with the total number of dots representing the difference in the number of trimers between Lab and Field thylakoids: for every 100 trimers in Lab thylakoids there are 70 trimers in Field thylakoids. Assuming three possible trimer combinations of LHCB1/2/3: (i) 2(LHCB1) + LHCB3 (blue dots, mostly M‐trimers), (ii) 2(LHCB1) + LHCB2 (red dots, mostly trimers capable of performing state transitions) (Pietrzykowska et al., 2014), and (iii) trimers containing only LHCB1 (green dots), Figure 3c shows that the Field thylakoids have very similar numbers of M‐trimers and a modest reduction in the number of trimers containing LHCB2. The main difference is the number of trimers containing only LHCB1 (green dots) is almost halved in the Field thylakoids.

We identified and quantified all six LHCI proteins occurring in Arabidopsis (Figure 3d). Despite a reduction in the number of PSI core complexes (Figure 3a), there was no significant change in the relative abundance of LHCA1, LHCA2 and LHCA4. This result is consistent with the immunoblot analysis reported by Mishra et al. (2012), where most of the LHCA proteins stay constant in Field compared to Lab conditions. We identified only one LHCI isoform, LHCA3, which decreased in abundance in the Field plants in line with PSI by 20%. In our previous analysis of laboratory plants grown under low, moderate and high light intensity we did not identify LHCA5 and LHCA6 (Flannery et al., 2021), which are low abundance isoforms known to mediate interactions with the NADH dehydrogenase‐like (NDH) complex in Arabidopsis (Peng et al., 2009; Yadav et al., 2017). In this study both were detected, with LHCA5 showing a significant 40% increase in the Field plants, while LHCA6 remained unchanged.

**FIGURE 4:**
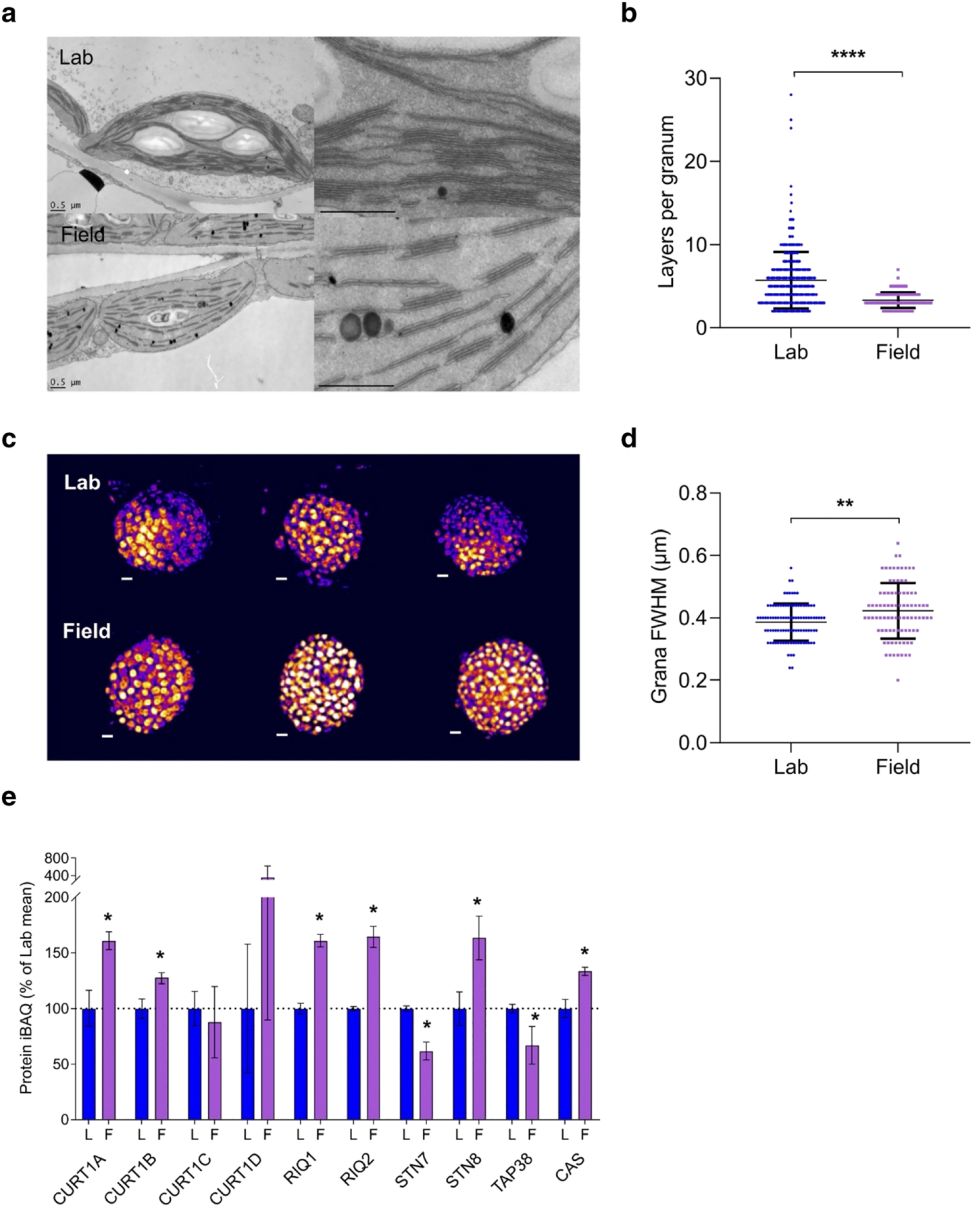
Image analysis of thylakoid architecture and MS‐base quantification of morphology‐related proteins from Arabidopsis plants cultivated in the lab and field. (a) Thin‐section electron micrographs of Lab (top row) and Field (bottom row) chloroplasts (scale bars: .5 μm) within leaves. (b) Number of membrane layers per grana stack calculated from electron microscopy images of chloroplasts in Lab (*n* = 354 grana stacks) and Field (*n* = 317 grana stacks) (Welch's *t* test. ****P < .0001). Error bars indicate mean ± SD. (c) Three‐dimensional structured illumination microscopy (3D‐SIM) images (shown as max projections on the z‐axis with tricubic sharp interpolation) of chloroplasts in Lab (top row) and Field (bottom row) leaves. (d) Full width at half‐maximum (FWHM) fluorescence intensity of the fluorescent spots (grana) in 3D‐SIM images of chloroplasts in Lab (*n* = 100) and Field (*n* = 88) plants (Welch's *t* test. **P < .01). Error bars indicate mean ± SD. (e) Relative abundances of proteins involved in the modulation of thylakoid membrane architecture, expressed as a percentage of the mean in Lab thylakoids. Details of sampling and results representation are as stated in Figure 3

Field grown Arabidopsis thylakoids possess fewer membrane layers per stack, a wider grana diameter and increased abundance of STN8, CAS, CURT1A, CURT1B, and RIQ1A, RIQ1B. Since a major determinant of grana stacking is LHCII‐LHCII interactions between membrane layers (Day et al., 1984), we next investigated how the changing composition of LHCII trimers in Field plants affects the number of membrane layers per granum using thin section electron microscopy (Figure 4a). Qualitatively, the chloroplasts of the Lab plants appeared more densely packed with thylakoid membranes than those of the Field plants, in which large expanses of stroma free of membranes were observed. Indeed, granal stacking was significantly (*P* < .0001) decreased in the chloroplasts from Field plants with dramatically fewer membrane layers per granum (Figure 4b). The number of membrane layers per grana in Field chloroplasts was also less variable than in the Lab chloroplasts, with no visible grana comprising more than 8 layers. This difference was also observed by Pribil et al. (2018), where growth under natural light conditions caused a 2‐ to 5‐fold reduction in grana height, and may be a result of the reduction in the number of LHCB1‐only trimers (Figure 3b) since LHCB1 contributes more significantly to stacking (Pietrzykowska et al., 2014). In contrast with acclimation to high light under laboratory conditions, where a decrease in the number of membrane layers is accompanied by a smaller grana membrane diameter (Flannery et al., 2021), here structured illumination microscopy showed chloroplasts from the Field had a larger diameter (*P* < .01) compared to Lab plants (Figure 4c,d).

In addition to LHCII isoform composition, the curvature inducing thylakoid proteins CURT1A/B/C/D and the reduced induction of non‐photochemical quenching (RIQ1 and RIQ2) proteins also strongly influence thylakoid stacking. Indeed, while Arabidopsis mutants lacking CURT1A/B/C/D have far fewer membrane layers per granum and a much wider grana diameter, mutants lacking RIQ1/2 show the opposite phenotype (Armbruster et al., 2013; Yokoyama et al., 2016). Pribil et al. (2018) previously found that, when grown in the natural environment, Arabidopsis mutants lacking all four CURT1 isoforms were significantly impaired in PSII efficiency, as measured by chlorophyll fluorescence analysis, compared to wild‐type plants, consistent with a 2‐fold increase in the abundance of CURT1A, B, and C in field‐grown plants compared to those grown in a controlled environment. In line with this finding, our analysis showed that Field plants had around 60% more CURT1A and 25% more CURT1B compared to Lab plants (Figure 4e). However, we observed no significant increase in CURT1C, in contrast to Pribil et al. (2018) nor in CURT1D. According to our analysis, both RIQ1 and RIQ2 increased by around 60% in Field plants (Figure 4e) in line with CURT1A and with the changes observed during acclimation to constant high light (Flannery et al., 2021).

Phosphorylation of LHCII and PSII by the STN7 and STN8 kinases, respectively, decreases grana stacking by increasing electrostatic repulsion on the stromal side of the membrane while dephosphorylation by TAP38 increases stacking (Armbruster et al., 2013; Fristedt, Willig, et al., 2009; Hepworth et al., 2021; Samol et al., 2012; Wood et al., 2019). Unexpectedly, although STN7 and STN8 both increase with growth light intensity under laboratory conditions (Albanese et al., 2018; Flannery et al., 2021), our analysis shows contrasting behavior of STN7 and STN8 in the field (Figure 4e). STN8 was 60% more abundant in the Field plants, whereas STN7 and its partner phosphatase, TAP38, both decreased by around 40% (Figure 4e). The lower relative abundance of these enzymes controlling phosphorylation of LHCII, as well as the decrease in the amount of LHCB2 (Figure 3b), are consistent with the loss of the PSI‐LHCI‐LHCII supercomplex observed in the BN‐PAGE gel (Figure 2a). CAS, a regulatory calcium sensor which also promotes dephosphorylation of LHCII (Cutolo et al., 2019), is increased by 25% in Field plants (Figure 4e). This is a smaller increase in relative abundance compared to that observed in controlled high light (70%) (Flannery et al., 2021) which may reflect a reduced need for LHCII dephosphorylation in Field plants since there is less STN7.

### 3.4 Field grown Arabidopsis plants show increased abundance of proteins associated with the regulation of light harvesting and electron transfer

**FIGURE 5:**
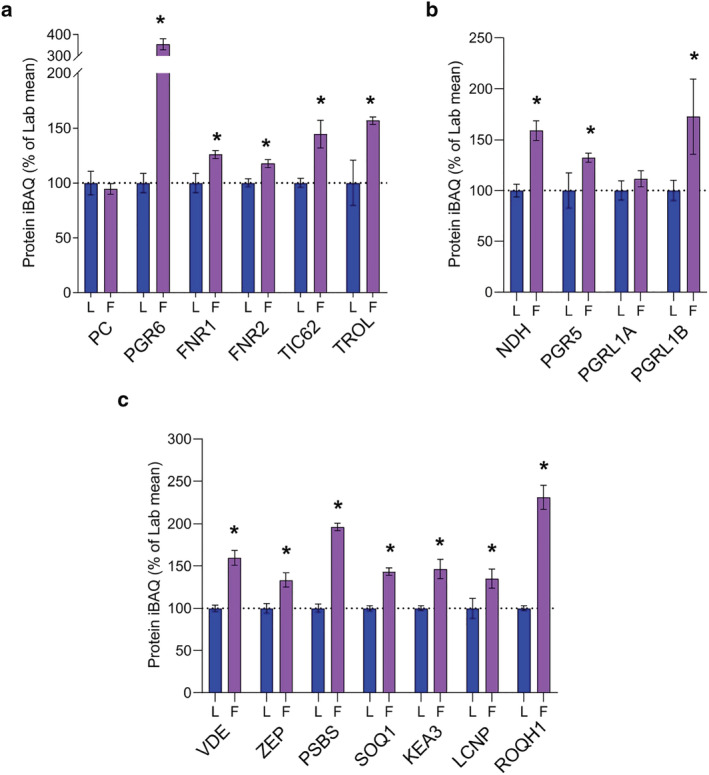
MS‐based quantification of thylakoid proteins involved in the regulation of electron transfer and light harvesting from Arabidopsis plants cultivated in the lab and field. (a) Relative abundance of key electron transfer proteins. (b) Relative abundance of CET‐related proteins. (c) Relative abundance of NPQ‐related proteins. Details of sampling and results representation are as stated in Figure 3

Previously, acclimation to high light under controlled laboratory conditions has been associated with increases in many of the proteins involved in linear electron transfer (LET) (Schöttler & Tóth, 2014; Walters, 2005). Here we found that the relative abundance of plastocyanin (PC), the electron donor for PSI, did not significantly change between Lab and Field plants (Figure 5a), in contrast to its reported increase in plants acclimated to high light in the laboratory (Albanese et al., 2018; Flannery et al., 2021). However, since PSI decreases in abundance in the Field plants, the PC/PSI ratio is still increased. PGR6, the plastoglobule‐associated regulator of the photoactive plastoquinone (PQ) pool (Pralon et al., 2019) whose relative abundance increases with growth light intensity, increased dramatically (+250%) in Field plants, much larger than the 100% increase previous seen upon high light acclimation in the laboratory. Acclimation to cold enhances the resistance of plants to photoinhibition by increasing the amount of PQ relative to PSII (Gray et al., 1996; Huner et al., 2008). Therefore, this difference in the magnitude of the response of PGR6 is consistent with the lower temperatures experienced by the Field plants. The relative abundance of FNR1 and FNR2 increased to a lesser extent (25% and 20%, respectively) in Field plants compared to controlled high light (Figure 5a) where they increased by ~50% (Flannery et al., 2021).

TIC62 and TROL have recently been proposed to regulate the efficiency of cyclic electron transfer (CET) through regulation of FNR tethering to the thylakoid membrane (Kramer et al., 2021). However, while TIC62 abundance correlated with an increased capacity for CET in laboratory high light acclimated Arabidopsis, TROL levels were unchanged (Flannery et al., 2021). Here, in contrast, the relative abundance of both TIC62 and TROL were increased by 40–50% (Figure 5a). Further indications of an increased capacity of Field plants for CET are provided in Figure 5b. There is a significant upregulation of NDH (60%), consistent with previous studies showing the importance of CET for rapid induction of PSI oxidation (photosynthetic control) in natural and laboratory fluctuating light conditions to avoid photo‐oxidative stress (Kono et al., 2017; Shimakawa & Miyake, 2018). An alternative route to NDH for electrons from ferredoxin to re‐enter the electron transfer chain during CET is via the PGR5/PGRL1‐dependent pathway (Buchert et al., 2020; Hertle et al., 2013). Here we find that PGR5 and PGRL1B increase in abundance by 30% and 70%, respectively, in Field plants (Figure 5b); less than the respective 60% and 150% increases seen in plants acclimated to constant high light (Flannery et al., 2021). The behavior of PGRL1A differed to that of PGRL1B, the former remaining constant in Field compared to Lab plants in all light environments analyzed. This corroborates recent results showing that, despite their close homology (DalCorso et al., 2008), expression of the two PGRL1 isoforms is differentially regulated, suggesting they may fulfill different roles in CET regulation (Flannery et al., 2021; Jin et al., 2017) and consistent with the observation that only PGRL1A is phosphorylated by STN8 (Reiland et al., 2011).

Under high light conditions the build‐up of ΔpH leads to the protonation of PSBS and violaxanthin de‐epoxidase (VDE), which converts the LHCII‐bound carotenoid violaxanthin to zeaxanthin. Together, PSBS and VDE induce a conformational change in LHCII which triggers qE, the major component of NPQ, allowing plants to dissipate excess absorbed excitation energy as heat in the PSII antenna (Ruban et al., 2012). Previous work showed that qE is more much important for plant fitness under naturally fluctuating light conditions in the field than in high light per se (Külheim et al., 2002; Li et al., 2000). MS analysis was used here to determine the relative abundance of proteins involved in the short‐term regulation of light harvesting in Arabidopsis from the Lab and the Field plants (Figure 5c). This analysis shows Field plants have a higher level of both VDE and zeaxanthin epoxidase (ZEP), which converts zeaxanthin back to violaxanthin in the reverse transition to the light harvesting state. Figure 5c shows that the relative abundance of VDE increases to a greater extent (60%) than ZEP (30%), with both proteins responding differently to field conditions than to acclimation to constant light intensities as described in Flannery et al. (2021). Constant high light acclimation did not affect the relative abundance of VDE, whereas ZEP increased by around 50% (Figure 5c), suggesting VDE is more important for fluctuating light than for constant high light irradiance where long term acclimation has reduced the need for rapid initiation of quenching. According to the MS analysis, PSBS increases 2‐fold in the field, approximately aligning with the 1.34‐fold increase previously determined for outdoor grown Arabidopsis by immunoblotting (Schumann et al., 2017). The difference in the relative abundances of the proteins VDE, ZEP and PSBS is consistent with the observation of Mishra et al. (2012), that field‐grown plants have an enhanced capacity for NPQ.

The K^+^/H^+^ antiporter KEA3 responds to sudden reductions in light intensity by releasing protons into the stroma, speeding up the return of LHCII to its light harvesting state in fluctuating light (Armbruster et al., 2014). KEA3 showed a 45% increase in Field compared to Lab plants (Figure 5c), whereas no increase is seen in high light acclimated Lab plants (Flannery et al., 2021), suggesting a particular importance for KEA3 under fluctuating light conditions. While much work has been done to study quenching involving PSBS and zeaxanthin, less is known about the sustained slowly relaxing form of NPQ (qI), part of which involves SOQ1, ROQH1, and LCNP (Amstutz et al., 2020; Brooks et al., 2013; Malnoë et al., 2018). SOQ1 and ROQH1 both function to suppress qI, whereas the chloroplastic lipocalin LCNP promotes this sustained form of quenching (Malnoë et al., 2018). Therefore, it is interesting that Field thylakoids contain elevated levels of all of these proteins (Figure 5c), a result which differs from that seen in constant light acclimation described in Flannery et al. (2021). Constant low light acclimated plants appear to increase their capacity for qI, observed as the upregulation of LCNP, whereas high light acclimated plants suppress it by increasing their levels of SOQ1 and ROQH1 (Flannery et al., 2021).

### 3.5 Upregulation of PSII repair machinery in the field‐grown plants

**FIGURE 6:**
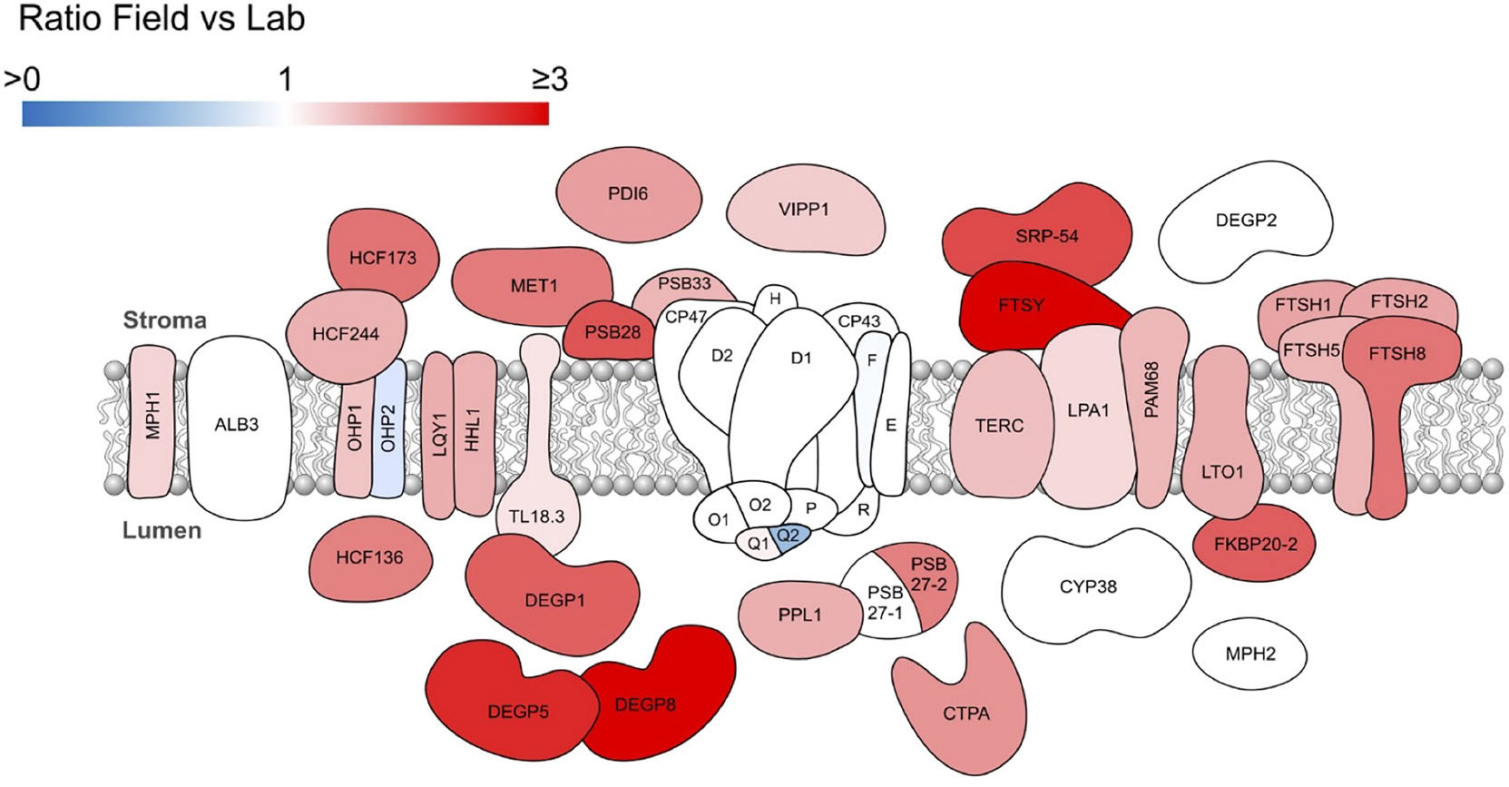
Schematic summary of the relative abundances of thylakoid proteins involved in the PSII repair cycle. This figure summarizes the MS‐based quantification results detailed in Figure S1. Proteins colored in shades of red are upregulated in Field relative to Lab thylakoids. The converse is shown by proteins colored in shades of blue, proteins that were not significantly different (*q* > .05) are not colored

The PSII reaction center D1 protein is known to be prone to photo‐oxidative damage particularly under high light and therefore an extensive repair machinery exists to mediate D1 excision and replacement (reviewed in Theis & Schroda, 2016). The relative abundance of STN8, which phosphorylates PSII to initiate its repair cycle (Järvi et al., 2015; Nath et al., 2013; Tikkanen et al., 2008), was increased by 60% under Field conditions (Figure 4e). Indeed, the relative amounts of many of the proteins of the PSII repair machinery, including MPH1, HCF173, HCF244, OHP1, HCF136, LQY1, HHL1, MET1, TL18.3, DEGP1, DEGP5, DEGP8, PSB28, PSB33, VIPP1, PPL1, PSB27‐2, FTSY, SRP‐54, TERC, LPA1, PAM68, LTO1, FKBP28.2, FTSH1, FTSH2, FTSH5, and FTSH8 (Figures 6 and S1) are increased in the Field versus Lab plants suggesting the increased importance of the cycle under natural conditions. Unexpectedly, the lumenal protein MPH2, which has a putative role in PSII disassembly during repair (Liu & Last, 2017), remained unchanged as did the immunophilin CYP38, which negatively regulates phosphatase activity on the PSII core (Vener et al., 1999). In this analysis, the detection of multiple isoforms of proteins with different behavior is notable. One possible model of PSII repair (Weisz et al., 2019) involves the storage of PSII subunits CP47, CP43, PSBH, several lower molecular weight subunits and the assembly factor PSB27 in a stable complex lacking a reaction center to avoid harmful photochemical reactions during the repair cycle. The identification of two isoforms of PSB27, only one of which (PSBP27‐2) is upregulated in Field plants, implies varied roles or regulation of this factor (Figure 6). The single‐transmembrane helix proteins OHP1 and OHP2 have both been shown to interact with the PSII biogenesis factor HCF244 and the PSII reaction center to form a complex that facilitates the co‐translational assembly of de novo synthesized D1 (Hey & Grimm, 2018; Li et al., 2019). While OHP1 displays the expected increase in Field plants (Figure 6), OHP2 appears to decrease in abundance. This observation contradicts previous evidence that OHP2 increases its expression in response to high light intensity (Andersson et al., 2003), as experienced under our field conditions. With the further finding that OHP2 associates with PSI (Andersson et al., 2003), its decrease in relative abundance in Field thylakoids seen in our analysis might be explained by its expression mirroring the decrease in PSI (Figure 3a). Analysis of an Arabidopsis mutant lacking OHP1 indeed supports roles for this protein in assembly of both PSII and PSI (Myouga et al., 2018).

### 3.6 Proteins specific for acclimation to a fluctuating natural light environment

**FIGURE 7:**
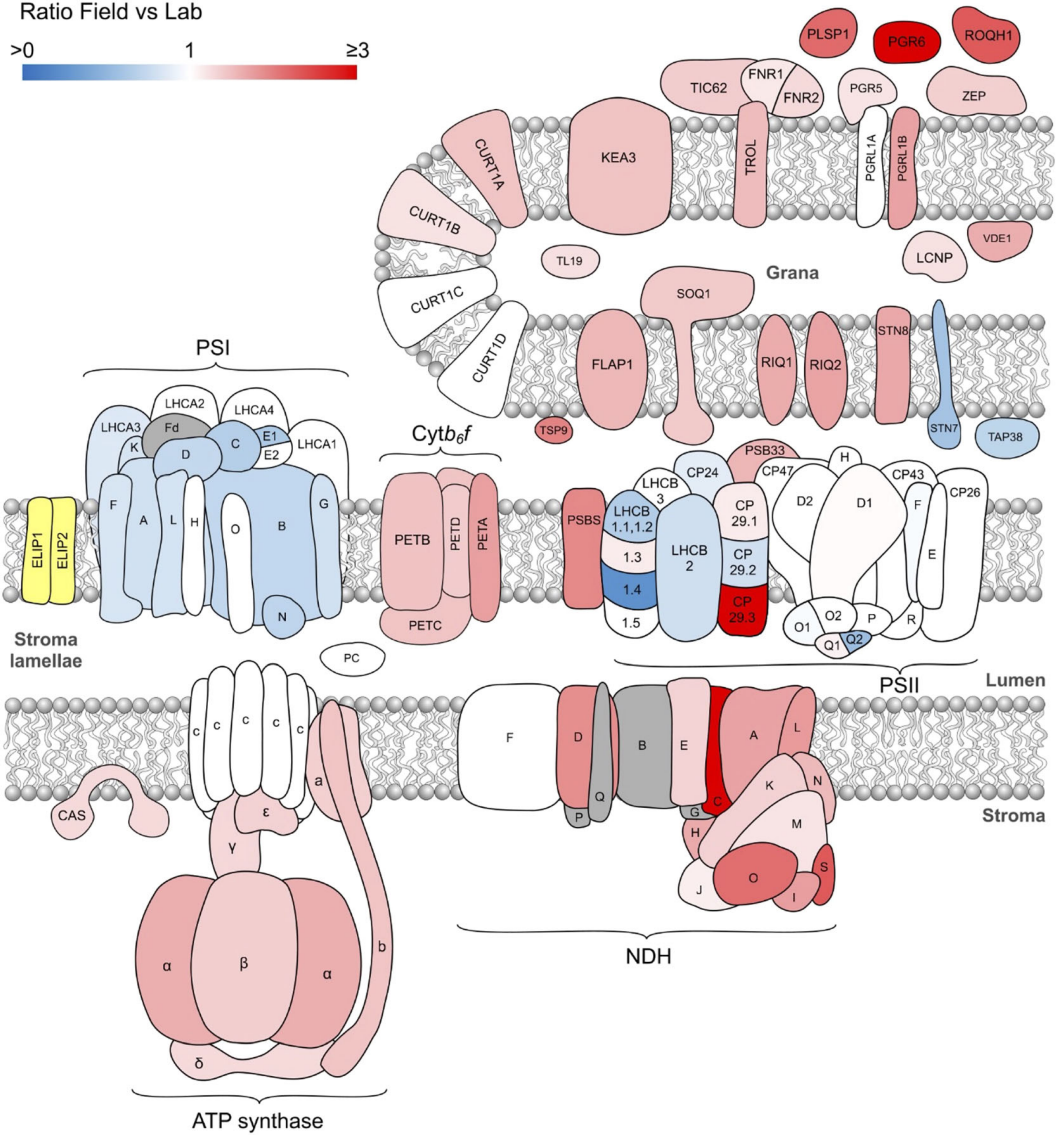
Schematic summary of the relative abundances of all thylakoid proteins considered in this study. Proteins are colored according to the criteria stated in Figure 6. In addition, proteins only detected in Field thylakoids are yellow and those not identified by our MS analysis are gray

Previously, it has been shown that the early light induced proteins (ELIPs) are either significantly upregulated or only detectable in plants grown in a natural light environment (Mishra et al., 2012; Norén et al., 2003). The expression of ELIPs is also upregulated in response to low temperature (Norén et al., 2003). Our analysis confirms these findings with the identification of both ELIP1 and ELIP2 exclusively in Field thylakoids (Figure 7). Constant high light and low temperature causes accumulation of ELIP1 and ELIP2 in wild type but does not cause a marked phenotype in mutants lacking these proteins (Rossini et al., 2006). Although the precise biological function or mechanism of these proteins is not currently known, ELIPs may function to prevent photo‐oxidative damage in high light stress through sequestration of free Chl molecules or stabilization of complexes during turnover of Chl‐containing proteins (Hutin et al., 2003), features which may be of increased relevance in the natural environment due to light and temperature fluctuations.

One notable protein that increased in abundance in Field thylakoids but not in response to constant light acclimation was TSP9, which showed a 2‐fold increase in the natural environment. TSP9 is found mostly in the grana and associates not only with LHCII but also with peripheral subunits of both PSII and PSI (Hansson et al., 2007). TSP9 is a phosphorylation target of STN7 and its absence detrimentally affects both state transitions and NPQ by an unknown mechanism. It has also been suggested that TSP9 facilitates the dissociation of antenna proteins from the PSII core under fluctuating light irradiance (Fristedt et al., 2008; Fristedt, Carlberg, et al., 2009). Therefore, the increased relative abundance of TSP9 in Field plants may align with reduced PSII‐LHCII supercomplex formation revealed by BN‐PAGE (Figure 2b). FLAP1 (fluctuating light acclimation protein 1), located in both the thylakoid membrane and chloroplast envelope, displayed a 50% increase in Field compared to Lab plants (Figure 7). FLAP1 has been implicated in the regulation of NPQ, since mutants lacking this protein have slightly higher levels of NPQ and a pale green phenotype, resulting from decreased leaf Chl content, under fluctuating light (Sato et al., 2017; Trinh et al., 2019).

### 3.7 Discussion

A principal aim of photosynthesis research is to inform strategies that enhance photosynthetic efficiency with focus on increasing yield in crops. With field cultivation, crops are subject to the variations and stresses of the natural environment, therefore it is vitally important to place our current understanding of photosynthetic mechanisms in the context of the field. Comparisons of Lab‐ and Field‐grown plants may reveal photosynthetic processes that are specifically relevant for crops and, therefore, establish promising new directions for research. Indeed, the recent finding that Arabidopsis plants grown under natural conditions show an acclimation strategy that is distinct from that seen for growth under constant high light under laboratory conditions (Schumann et al., 2017) highlights the importance of such work. Although Arabidopsis is not an agriculturally relevant species, the time and location used for the outdoor growth of the plants in this study were aimed at replicating field conditions; the plants were watered regularly to mimic irrigation, grown during summer, and with minimal shading from buildings or canopy. Previous work comparing gene expression in Arabidopsis plants grown under different artificial light environments (Seiler et al., 2017), including fluorescent tubes and LEDs of various intensities and spectral qualities, demonstrated that spectral composition affected mRNA populations relating to a wide range of cellular processes. However, the observed gene expression changes, determined at the transcriptomic level, were only loosely associated with phenotype, suggesting the need for a proteomic analysis. Indeed, previous comparisons of various metrics have found surprisingly modest associations between transcriptome and proteome, with non‐significant correlation (*R* = .186) in yeast (Foss et al., 2007; Fu et al., 2009). Furthermore, at least 80 proteins are encoded on the chloroplast genome of Arabidopsis with many, including *psbA* (PSII D1 subunit) *and rbcL* (Rubisco large subunit), that are regulated at the translational level (Chotewutmontri & Barkan, 2018; Sun & Zerges, 2015). Taken together, these observations underline the importance of directly measuring protein abundance. In this study using label‐free protein quantification provided new insights into how the abundance of an extended range of thylakoid proteins changes in Field versus Lab plants. Curiously, the Field plants appear to incorporate aspects of the laboratory acclimation response to both low light, such as increases in ZEP, LHCB1.3, CP29.1 and LCNP, and high light, such as increased levels of the cyt*b*_6_ *f*, ATP synthase and FNR1 and FNR2 (Flannery et al., 2021). The thylakoid proteome of Field plants also included proteins not identified under laboratory conditions such as FLAP1 and the ELIP1 and 2. The distinct proteome of the Field plants suggests that the combination of fluctuating temperature and light intensity in the natural environment presents a unique set of challenges.

Among the most prominent of the proteomic changes observed in Field plants was the altered abundance of light‐harvesting proteins, suggesting a different light‐harvesting strategy is required compared to controlled laboratory conditions. The 30% decrease in the relative abundance of LHCII seen in the Field plants was mostly derived from the LHCB1 containing peripheral L‐trimers, which serve as a peripheral antenna to PSII (Pietrzykowska et al., 2014). This decrease was corroborated by the smaller fluorescence excitation cross‐section for PSII, with a lower contribution at the Chl *b* wavelengths typical of LHCII. These changes are a similar, though more extreme, version of the laboratory high light acclimation response and can be understood as a shift from light to electron transfer limitation on the photosynthetic light reactions (reviewed by Schöttler & Tóth, 2014; Walters, 2005). The observed decrease in the PSI/PSII ratio and PSI‐LHCI‐LHCII supercomplex abundance in Field plants suggests a shift to State I conditions, where dephosphorylated LHCII is mostly coupled to PSII. This result contrasts with the study of Wientjes et al. (2013a) who observed that Arabidopsis plants adopted State II, where phosphorylated LHCII is energetically coupled to PSI, under natural sunlight. The decreased amounts of the STN7 and TAP38 proteins associated with state transitions also support the view that PSI is less likely to be light limited under the natural conditions experienced in this experiment—a contrast to the study of Mishra et al. (2012), who reported that Field plants were capable of performing state transitions at a similar level to indoor plants. Since the ratio o f far‐red light (>700 nm) absorbed exclusively by PSI would likely be similar in these three studies, the explanation for the discrepancy probably lies in the differing light intensities experienced by the plants, i.e. most days below 548 μmol m^−2^ s^−1^ in the Wientjes et al. (2013a) and Mishra et al. (2012) studies compared to most days above this intensity here. Since dephosphorylation of LHCII is promoted by high light (Mekala et al., 2015; Rintamäki et al., 1997) the result is a shift to State I. This shift in the Field plants is also corroborated by their increased grana diameter relative to Lab plants, since dephosphorylation of LHCII promotes this effect (Hepworth et al., 2021; Wood et al., 2019). Indeed, decreased LHCII phosphorylation and reduction in the levels of LHCB1 containing trimers may overrule the increased relative abundance of CURT1A and CURT1B in Field plants that otherwise would be anticipated to increase the number of membrane layers per granum (Armbruster et al., 2013; Pietrzykowska et al., 2014). Likewise, the increased relative abundance of RIQ1 and RIQ2, which negatively correlate with number of membrane layers per grana (Yokoyama et al., 2016), may also contribute to this effect. The combination of decreased grana stacking in the Field plants with increased grana diameter is in contrast to that seen in laboratory high light conditions where both parameters decrease, consistent with retention of State II in these circumstances (Flannery et al., 2021). A larger grana diameter may be advantageous to Field plants protecting them from high light induced damage through promotion of PSI oxidation (photosynthetic control) and CET through stricter partitioning of the electron carriers PC and PQ between grana and stromal lamellae thylakoid domains (Hepworth et al., 2021).

The shift away from light limitation to increase electron transfer flux in Field plants is also consistent with the increased relative abundance of cyt*b*_6_ *f*, FNR1 and FNR2, the two complexes with the highest flux control coefficients for the LET chain (Hajirezaei et al., 2002; Kirchhoff et al., 2000). Compared to the laboratory high light acclimation response, however, the increase in cyt*b*_*6*_ *f* in Field conditions was much larger and the increase in FNR1 and FNR2 smaller, suggesting the principal limitation is transfer through the chain rather than from chain to sink (Flannery et al., 2021). This finding may reflect the lower temperatures experienced by our Field grown plants since chilling stress is known to increase the levels of PGR6 and the size of the photoactive PQ pool, suggesting PQ diffusion may be hampered (Flannery et al., 2021; Gray et al., 1996; Huner et al., 2008). As with LET, an increased abundance of CET‐related proteins was also observed (LHCA5, NDH, PGRL1B, and PGR5) corroborating recent results showing the importance of CET under fluctuating light regimes (Kono et al., 2017; Shimakawa & Miyake, 2018; Suorsa et al., 2012). Increases in TIC62 and TROL in the Field plants may also serve to enhance CET by promoting FNR tethering to the thylakoid membrane in the vicinity of cyt*b*_6_ *f* (Kramer et al., 2021). We find however that the components of the NDH‐dependent CET pathway are most dramatically increased. Possibly under field conditions the higher H^+^/e^−^ ratio of NDH‐dependent CET (8), compared to the PGR5/PGRL1 pathway (H^+^/e^−^ = 4) means the former is favored to fulfill the increased requirement for ATP to sustain PSII repair and PSI biogenesis arising from environmental stress. This would be consistent both with the higher abundance of PSII repair proteins we observe and the larger amounts of ATP synthase (Figure 3a), the latter indicative of a higher proton flux due to increased coupled LET and CET. The other major difference compared to plants grown under controlled light conditions was the increased relative abundance of NPQ‐related proteins (Figure 5c). Unlike in high light acclimated conditions in the laboratory both VDE and ZEP increase in addition to PsbS (Albanese et al., 2018; Flannery et al., 2020) in the field grown plants. Since these three proteins are the principal modulators of qE‐kinetics (Ruban et al., 2012), this result suggests optimal growth in fluctuating light requires both speedier formation *and* relaxation from quenching, consistent with the study of Kromdijk et al. (2016). A response observed in the Field plants that is more typical of low light acclimation in the laboratory was the increased abundance of the LCNP protein (Malnoë et al., 2018). Upregulation of this protein, which modulates the ΔpH‐independent slowly relaxing form of NPQ, called qI, may reflect the need to protect PSII under low temperature conditions that suppress rapid formation of qE‐type quenching.

Overall, the results reported here demonstrate that Field‐grown Arabidopsis plants adopt a thylakoid proteomic composition that is distinct from that seen in Lab‐grown plants acclimated to either high or low light. The natural environment challenges the mechanisms that regulate the expression of key proteins involved in light harvesting and electron transfer in ways that controlled growth environments do not. To gain further insights into the regulatory mechanisms that underpin environmental acclimation, MS‐based quantitative proteomic analysis now must be employed to extend the exploration of photosynthesis‐related mutant strains beyond the laboratory and into the natural environment.

## AUTHOR CONTRIBUTIONS

S.E.F., M.J.D., C.N.H., P.J.J., and M.P.J. designed the experiments. S.E.F., C.H., F.P., and W.H.J. performed the experiments. P.J.J., M.J.D., S.E.F., C.N.H., and M.P.J. wrote the manuscript. All authors proof‐read and approved the manuscript.

## CONFLICT OF INTEREST

The authors have no conflict of interest to declare.

## Supporting information


**Table S1.** **Relative abundance of thylakoid proteins calculated using label free quantitative proteomics.** Details of the quantified thylakoid proteins including functional category, protein name, description, UniProtKB identifier, and abundance ratios. Proteins with altered abundance in the field were identified by a modified Welch's t‐test (*q* < .05) implemented in Perseus (Tyanova et al., 2016) with a 5% permutation‐based FDR calculated from 250 randomizations. Median protein iBAQ (Cox & Mann, 2008; Schwanhüusser et al., 2011) values were used for the calculation of ratios in the Field versus the Lab.

**Table S2.** **Intensity‐based absolute quantification (iBAQ) of *Arabidopsis* thylakoid proteins.** MS‐identified protein iBAQ (Cox & Mann, 2008; Schwanhüusser et al., 2011) values following normalization to the intra‐analysis sum of key photosynthetic complexes PSII (PSBA, PSBB, PSBC, PSBD, PSBE, PSBF, PSBH, PSBO1, PSBO2, PSBP1, PSBP2, PSBQ1, PSBQ2, PSBR), PSI (PSAA, PSAB, PSAC, PSAD, PSAE1, PSAE2, PSAF, PSAG, PSAH, PSAK, PSAL, PSAN, PSAO), Cyt*b6f* (PETA, PETB, PETC, PETD), and ATP synthase (ATPA, ATPB, ATPC, ATPD, ATPE, ATPF, ATPH, ATPI) with supporting information generated from MaxQuant (Cox & Mann, 2008): number of peptides identified, protein sequence coverage, identification score (derived from peptide posterior error probabilities), and MS/MS count. Identifying information from the *Arabidopsis thaliana* UniProtKB proteome database is given as majority protein IDs, protein names, and gene names.

**Figure S1.** **A‐D**, MS analysis showing the relative abundance of proteins involved in PSII repair, expressed as a percentage of the mean in Lab thylakoids. Sampling details are as stated in Figure 3.

## Data Availability

The mass spectrometry proteomics data have been deposited to the ProteomeXchange Consortium via the PRIDE partner repository (http://proteomecentral.proteomexchange.org) with the data set identifier PXD027391. All other data can be obtained from the corresponding authors upon request. The following figures have associated raw data: Figures 3, 4, 5, 6, 7. Quantitative MS analysis results are provided in Tables S1 and S2.
