# Supplementary figures and images for: Comparative proteomics of thylakoids from Arabidopsis grown in laboratory and field conditions
Source: Plant Direct. 2021 Oct 20;5(10):e355. doi: 10.1002/pld3.355 (PMC8528093; doi:10.1002/pld3.355)

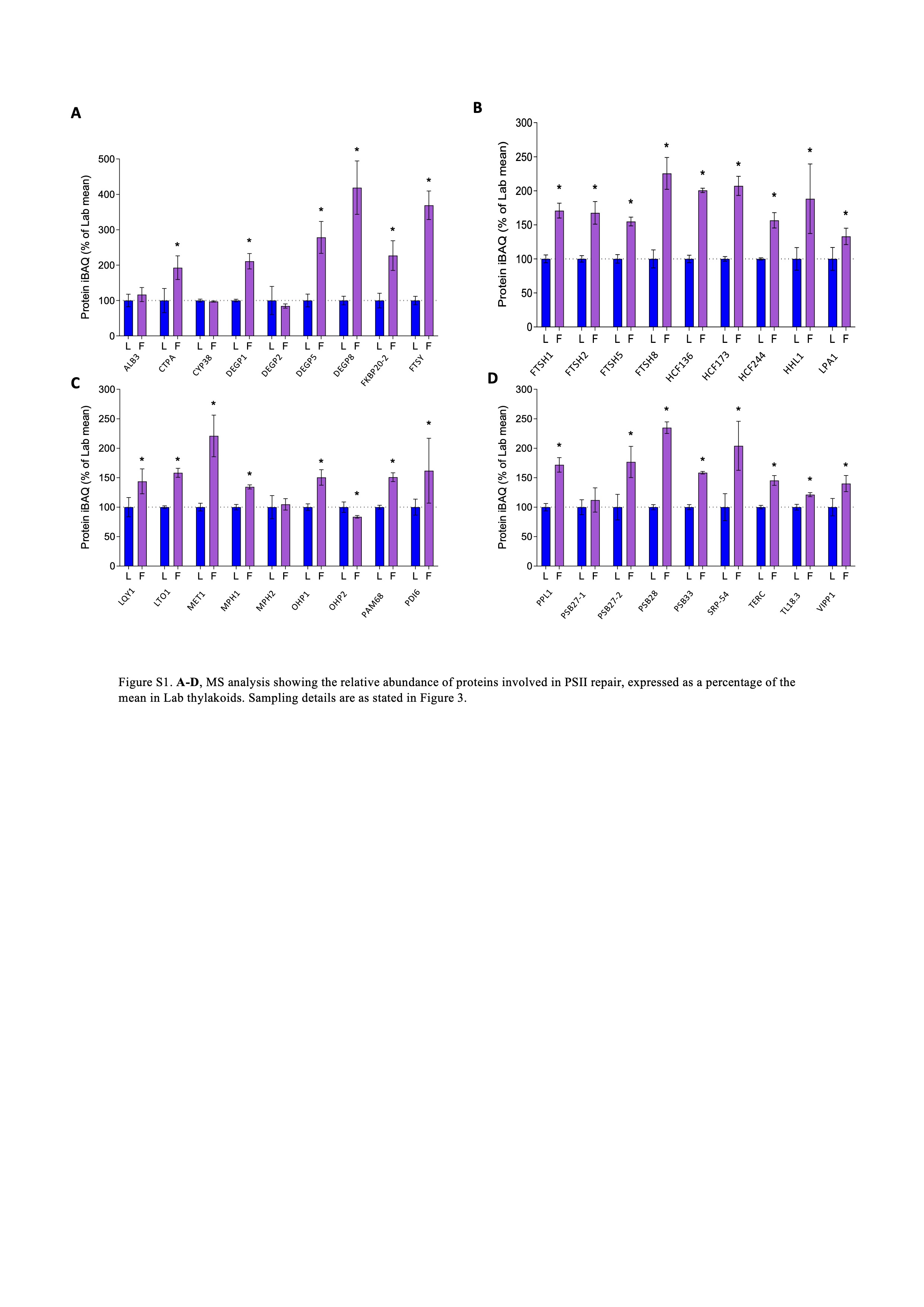

Supplement: Supplementary file 3 — Figure S1. A‐D, MS analysis showing the relative abundance of proteins involved in PSII repair, expressed as a percentage of the mean in Lab thylakoids. Sampling details are as stated in Figure 3. [file PLD3-5-e355-s003.jpg]
